# Epidemiology, risk factors, maternal and perinatal outcomes of uterine rupture: a 7-year retrospective study from a Moroccan tertiary hospital

**DOI:** 10.3389/fgwh.2026.1814132

**Published:** 2026-07-22

**Authors:** Hanane Houmaid, Hafssa Naji, Myriem Sali, Karam Harou, Bouchra Fakhir, Lahcen Boukhanni, Hamid Asmouki, Abderraouf Soummani

**Affiliations:** Gynecology Obstetrics Department, Mohammed VI University Hospital, Cadi Ayyad University, Marrakesh, Morocco

**Keywords:** uterine rupture, scarred uterus, unscarred uterus, maternal outcomes, perinatal outcomes, low-resource settings, neglected uterine rupture

## Abstract

**Background:**

Uterine rupture (UR) is a rare but life-threatening obstetric emergency associated with high maternal and perinatal morbidity and mortality, particularly in low-resource settings. Delayed diagnosis and management significantly worsen outcomes. This study aimed to describe the epidemiological characteristics, risk factors, management, and maternal and perinatal outcomes of uterine rupture, with particular emphasis on distinguishing neglected from non-neglected cases.

**Methods:**

A retrospective descriptive study was conducted at Mohammed VI University Hospital, Marrakesh, Morocco, between January 2017 and December 2024. All confirmed cases of uterine rupture were included. Demographic, obstetric, clinical, and surgical data were collected from medical records. Maternal and perinatal outcomes were compared according to uterine scar status and delay in management.

**Results:**

Among 115,121 deliveries, 74 cases of uterine rupture were identified, corresponding to an incidence of 0.06%. Rupture occurred in unscarred uteri in 57% of cases and in scarred uteri in 43%. Inadequate or absent antenatal care was reported in 81% of patients. Hysterectomy was required in 23% of cases, predominantly in neglected ruptures. Maternal mortality was 1.4%, while perinatal mortality reached 35.1%, occurring mainly in neglected and unscarred uterine ruptures.

**Conclusion:**

Uterine rupture remains a severe and largely preventable obstetric emergency in low-resource settings. Strengthening antenatal care, eliminating harmful obstetric practices, and ensuring timely referrals and intervention are essential to reduce preventable morbidity and mortality.

## Introduction

Uterine rupture (UR) is a rare but catastrophic obstetric complication characterized by a full-thickness disruption of the uterine wall, involving the endometrium, myometrium, and serosa, resulting in direct communication between the uterine cavity and the peritoneal space (1, 2). It can occur during pregnancy, labor, or the immediate postpartum period and is associated with severe maternal and perinatal morbidity and mortality. A related but distinct condition, uterine dehiscence, typically occurs in scarred uteri and is defined by incomplete separation of the uterine wall with preservations of the serosa, often remaining clinically silent (1).

Clinically, uterine rupture (UR) most commonly presents acute abdominal pain, uterine tenderness, vaginal bleeding, cessation of uterine contractions, and fetal heart rate abnormalities, particularly bradycardia (3). However, atypical or masked presentations—especially in women receiving analgesia—may delay diagnosis and worsen outcomes. Management requires immediate multidisciplinary intervention and emergency laparotomy, with surgical options ranging from uterine repair to hysterectomy, depending on the extent of the rupture and the patient's hemodynamic status as reported in previous clinical reviews (4, 5).

In high-income countries, uterine rupture (UR) is a rare event, with an estimated prevalence of approximately 0.006%in population-based studies from high-income countries. Most cases are associated with a trial of labor after cesarean delivery (TOLAC) or the inappropriate use of uterotonic agents such as oxytocin (6–8). Favorable outcomes in these settings are largely attributed to continuous intrapartum monitoring, timely diagnosis, and rapid access to emergency surgical care. Consequently, international guidelines, including those issued by the French College of Obstetricians and Gynecologists (CNGOF), emphasize strict selection criteria for TOLAC, standardized labor surveillance, and guaranteed immediate access to operative facilities (9).

In contrast, uterine rupture (UR) remains a significant public health issue in low- and middle-income countries (LMICs), where it disproportionately contributes to maternal and perinatal mortality. The global incidence of UR is estimated at approximately 0.07%, although rates as high as 1.3% have been reported in parts of Africa (10). According to the World Health Organization (WHO), Sub-Saharan Africa and South Asia account for more than 87% of global maternal deaths, with UR being a major contributing factor (11). Identified risk factors in these regions include grand multiparity prolonged or obstructed labor, inadequate antenatal care, delayed referral, inappropriate use of uterotonics, and harmful obstetric practices (12, 17).

Despite this burden, data on uterine rupture (UR) in North African countries, particularly Morocco, remain limited. The lack of national epidemiological data hinders the development of targeted preventive strategies and context-appropriate clinical guidelines. Furthermore, few studies have differentiated between promptly managed and neglected cases of UR, even though delays in diagnosis and treatment are known to critically affect maternal and neonatal outcomes.

In this context, the present study was conducted at the Mohammed VI University Hospital of Marrakech, Morocco, a tertiary referral center managing obstetric emergencies from both urban and rural areas. The objective of this study is to determine the prevalence of uterine rupture, analyze associated maternal and perinatal outcomes, and identify risk factors contributing to morbidity and mortality, with particular emphasis on differentiating neglected from non-neglected cases. By addressing these gaps, this study aims to provide evidence to inform preventive strategies, optimize intrapartum care, and improve maternal and neonatal outcomes in similar low-resource settings.

## Methods

### Study design and setting

This retrospective, descriptive study was conducted at the Mohammed VI University Hospital, department of Gynecology and Obstetrics, a tertiary care facility located in Marrakesh, Morocco. The investigation spanned a seven-year period, from January 1, 2017, to December 31, 2024.

### Study population

The study population comprised all women diagnosed with uterine rupture and managed at the hospitals during the specified timeframe. Cases were identified through a review of delivery records, surgical exploration, and patient medical charts. Inclusion criteria required confirmation of uterine rupture based on intraoperative findings or clinical diagnosis. Records lacking sufficient clinical information were excluded from the analysis.

### Variables and data collection

Data were extracted using a standardized collection form designed to capture pertinent clinical and demographic information from patient records. The primary outcome measures encompassed maternal outcomes—such as recovery status, duration of hospitalization, surgical interventions (including uterine repair and hysterectomy), requirement for blood transfusion, maternal mortality— and fetal outcomes, including live birth, stillbirth, and neonatal condition at discharge Due to substantial missing data in the medical records, Apgar scores and birth weights were not analyzed. Similarly, the anatomical site of uterine rupture was not consistently documented and could therefore not be reliably assessed. Secondary variables included socio-demographic factors (maternal age, parity, and residence), obstetric and labor characteristics (antenatal care attendance, history of cesarean section or uterine surgery, presence of obstructed labor signs, and hypovolemic shock upon admission), as well as management-related factors (type of surgical procedure performed and classification of uterine rupture as complete or incomplete). Maternal death attributable to uterine rupture was defined as any maternal mortality directly resulting from the rupture or its complications.

Due to substantial missing data in the medical records, Apgar scores and birth weights were not analyzed. Similarly, the anatomical site of uterine rupture was not consistently documented and could therefore not be reliably assessed.

### Data analysis

All data entry and analysis were conducted using Microsoft Excel. Descriptive statistics, including frequencies, percentages, means, and ranges, were used to summarize the study findings. Given the retrospective design and the limited sample size, the study was primarily descriptive in nature. Therefore, no multivariable analysis was performed, and comparisons between groups should be interpreted with caution.

### Definitions

Neglected uterine rupture was defined as a rupture diagnosed more than 24 h after symptom onset or occurring in the context of prolonged labor associated with delayed referral and delayed surgical management. Non-neglected uterine rupture referred to cases diagnosed and managed within a short interval after symptom onset.

## Results

Between 2017 and 2024, a total of 115,121 deliveries were recorded in our institution, among which 74 cases of uterine rupture were identified, corresponding to an incidence of 0.06%. Of these, 32 cases (43%) occurred in scarred uteri and 42 (57%) in unscarred uteri. Among the scarred uteri, 21 cases (65.6%) involved a single uterine scar and 11 (34.4%) multiple scars. The mean maternal age was 30years (range 19–46), and the mean parity was 3.36 (range 1–12). Primiparas accounted for 5.5% of cases, multiparas 70%, and grand multiparas 23%. Most patients (85.1%) were referred to as peripheral healthcare facilities, while only 14.9% were diagnosed in hospital. Annual case distribution ranged from 7 to 12 ruptures, with no clear decreasing trend over the study period (Table 1).

**Table 1:** Sociodemographic and obstetric characteristics of patients with uterine rupture (*N* = 74).

Variables	Frequency (n)	Percent (%)
Maternal age (years)		
<20	4	5.4
20–35	53	71.6
>35	16	21.6
Marital status		
Married	67	90.5
Single	5	6.8
Widowed/Divorced	1	1.4
Parity		
Primiparous	4	5.4
Multiparous (2–4)	52	70.3
Grand multiparous (≥5)	17	23
Gestational age at rupture		
20–27 weeks (very preterm)	3	4.1
28–36 weeks (moderate–late preterm)	6	8.1
≥37 weeks (term)	64	86.5
>41 weeks	0	0.0
Antenatal care (ANC)		
Adequate/complete	14	18.9
Inadequate	23	31.1
None	36	48.6
Mode of delivery		
Spontaneous vaginal delivery (SVD)	9	12.2
Instrumental delivery (forceps/vacuum)	12	16.2
Cesarean section (C/S)	52	70.3
Vaginal delivery after induction	0	0.0
Referral status		
Referred to peripheral facility	63	85.1
Diagnosed in-hospital	11	14.9
Clinical presentation		
Acute abdominal pain	60	81.1
Fetal distress/intra uterine fetal demise	47	63.5
Vaginal bleeding	34	45.9
Hemodynamic instability	18	24.3
Uterine condition		
Scarred uterus	32	43.2
Unscarred uterus	42	56.8
Hysterectomy		
Hysterectomy performed	17	23
Conservative surgical management	57	77
Blood transfusion (>2 units)	52	70.3
Neonataldeaths	26	35.1
Maternal death	1	1.4

### Clinical presentation

The most frequent presenting symptoms were acute abdominal pain (reported in 82% of cases), fetal distress and intrauterine fetal demise (65%), vaginal bleeding (47%), and hemodynamic instability (24%). The mean time from symptom onset to diagnosis was 5.6 h in promptly managed cases, while neglected ruptures were diagnosed after more than 24 h (Table 1).

### Pregnancy and delivery outcomes

**Figure 1:**
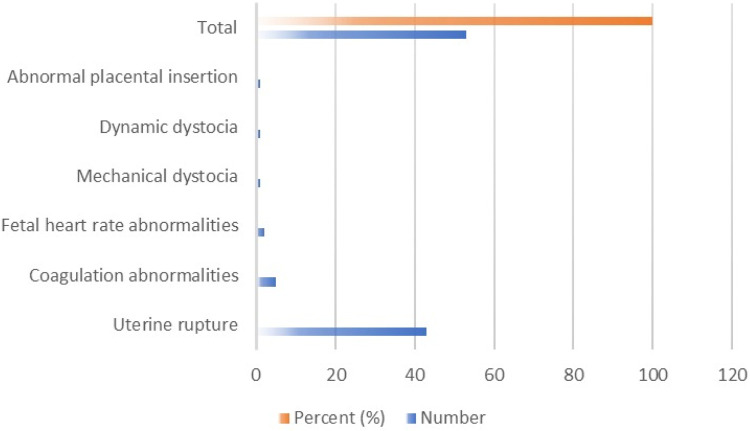
Distribution of indications for cesarean delivery in cases of uterine rupture.

Most pregnancies were full-term (87%), with 13% occurring preterm. Vaginal delivery was recorded in 29% of cases—including 12 instrumental deliveries—while 71% underwent cesarean section. Suspicion of uterine rupture was the primary indication for cesarean delivery (81%), followed by coagulopathy, fetal heart rate abnormalities, dystocia, and abnormal placental insertion (Figure 1). Antenatal care was inadequate for most patients: 49% had no prenatal care, 32% had insufficient monitoring. 19% received adequate follow-up.

### Maternal outcomes

Hysterectomy was required in 23% of cases, while 77% were managed conservatively. Blood transfusions exceeding two units were necessary in 71% of cases. One maternal death (1.4%) occurred due to septic shock in a neglected rupture, and other complications included bladder injury (2.7%), ureteral ligation (2.7%), stercoral peritonitis (1.4%), and acute renal failure (1.4%). Maternal complications and hysterectomy were also more frequent in unscarred ruptures (Table 3).

### Perinatal outcomes

There were 26 perinatal deaths (35%), predominantly among neglected ruptures and unscarred uteri. Neonatal mortality was higher in unscarred uteri (45%) compared with scarred uteri (22%) (Table 2).

**Table 2:** Maternal and perinatal outcomes according to delay in diagnosis.

Variable	Non neglected (*n* = 52)	Neglected (*n* = 22)	Scarred uterus (*n* = 32)	Unscarred uterus (*n* = 42)	Fundal pressure (*n* = 12)
Maternal complications	6 (11.5%)	5 (22.7%)	4 (12.5%)	7 (16.7%)	4 (33.3%)
Hysterectomy	9 (17.3%)	8 (36.4%)	5 (15.6%)	12 (28.6%)	5 (41.7%)
Perinatal mortality	11 (21.2%)	15 (68.2%)	7 (21.9%)	19 (45.2%)	7 (58.3%)
Mean diagnosis delay	6 h	>24 h	7 h	11 h	–

### Risk factors

**Figure 2:**
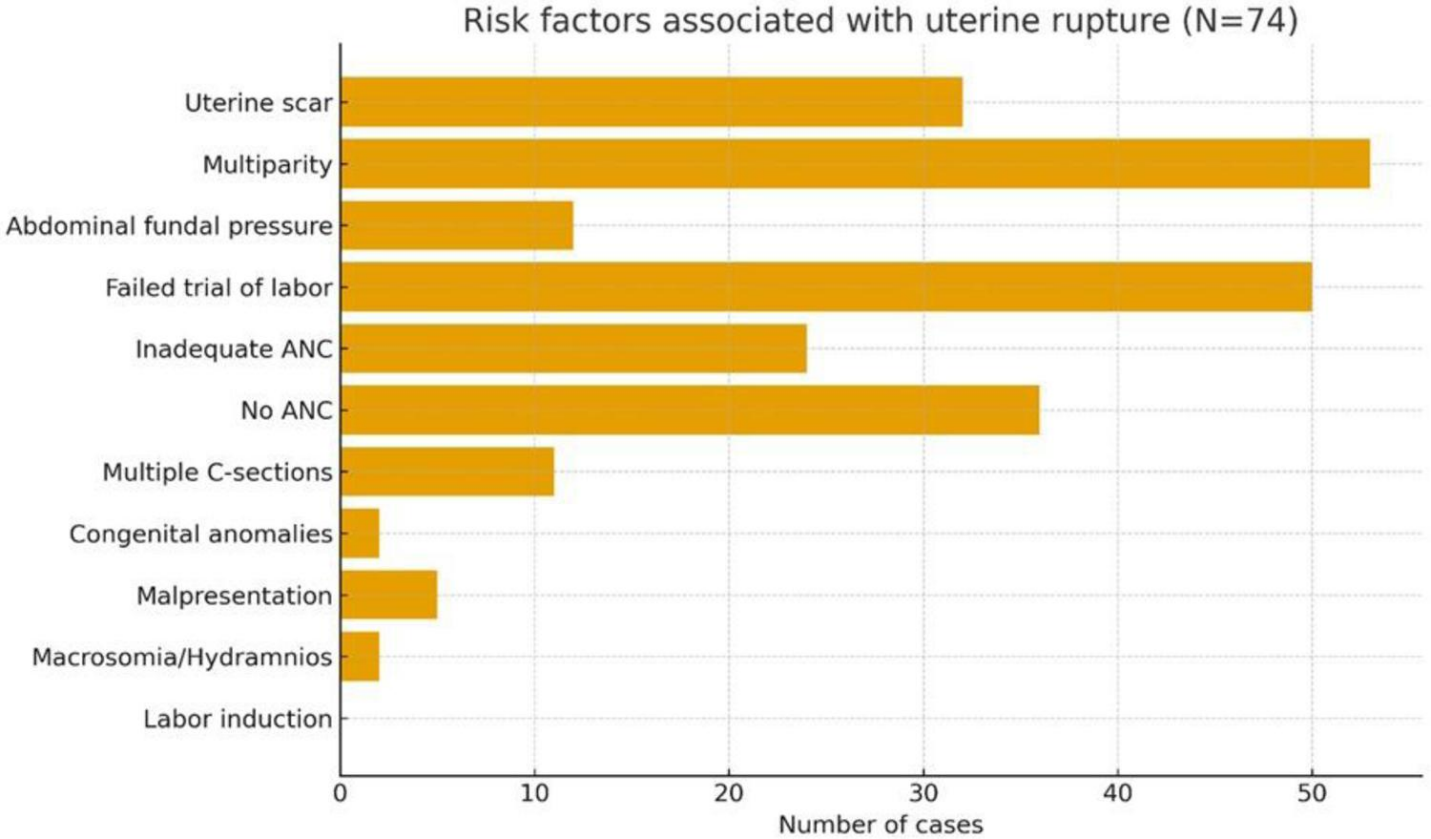
Frequency of risk factors associated with uterine rupture (*N* = 74).

The main risk factors identified were inadequate or absent antenatal care (81%), multiparity (71%), 146 failed trials of labor after cesarean (67%), uterine scar (43%), abdominal fundal pressure (17%), and multiple previous cesarean deliveries (15%) (Figure 2). Fundal pressure was predominantly associated with unscarred uteri and was observed more frequently alongside higher rates of hysterectomy and perinatal mortality. This emphasizes its role as a preventable and iatrogenic risk factor. Less frequent contributors included malpresentation (6.7%), congenital uterine anomalies (2.7%) and macrosomia/hydramnios (2.7%) (Figure 2).

### Comparative outcomes by scar status and delay in management

Non-neglected ruptures in scarred uteri were generally associated with lower maternal and perinatal morbidity, whereas neglected ruptures in unscarred uteri had the highest rates of hysterectomy (50%), neonatal deaths (87.5%), and even one maternal death (12.5%). These findings underscore the critical importance of timely diagnosis and intervention, particularly in unscarred uteri where the clinical course is more severe and prognosis is poorer. Blood transfusions, urinary injuries, and intensive care unit (ICU) stays were also more frequent in these cases (Table 3). Percentages were calculated based on the total number of cases (*N* = 74). For variables with incomplete information, missing data was explicitly reported.

**Table 3:** Comparative outcomes according to scar status and delay in management.

Variable	Scarred uterus—non-neglected (*n* = 26)	Scarred uterus—Neglected (*n* = 6)	Unscarred uterus—non-neglected (*n* = 34)	Unscarred uterus—Neglected (*n* = 8)
Hysterectomy	4 (15.4%)	1 (16.7%)	8 (23.5%)	4 (50.0%)
Maternal deaths	0	0	0	1 (12.5%)
Blood transfusion (>2 units)	18 (69.2%)	5 (83.3%)	22 (64.7%)	7 (87.5%)
Bladder injury	1 (3.8%)	0	1 (2.9%)	1 (12.5%)
Ureteral ligation	1 (3.8%)	0	1 (2.9%)	0
Stercoral peritonitis	0	0	1 (2.9%)	0
Acute renal failure	0	0	1 (2.9%)	0
Neonataldeaths	5 (19.2%)	2 (33.3%)	12 (35.3%)	7 (87.5%)
Multiparity	18 (69.2%)	4 (66.7%)	24 (70.6%)	7 (87.5%)
Inadequate or absent ANC	20 (76.9%)	5 (83.3%)	26 (76.5%)	8 (100%)

## Discussion

This study provides a detailed analysis of uterine rupture over a seven-year period in a Moroccan tertiary referral hospital and represents, to our knowledge, one of the few national series addressing this severe obstetric complication. Uterine rupture remains a rare but life-threatening event, with particularly serious maternal and perinatal consequences in low-resource settings, especially when diagnosis and management are delayed (1, 4, 5).

The incidence of uterine rupture observed in our study (0.06%) is consistent with rates reported in several hospital-based studies from low- and middle-income countries (10, 12, 17, 18). In contrast, population-based studies from high-income countries report substantially lower incidences, reflecting improved antenatal care, intrapartum surveillance, and timely access to emergency obstetric care (19, 21). This discrepancy highlights persistent inequalities in maternal healthcare systems worldwide.

More than half of uterine ruptures in our cohort occurred in unscarred uteri. This finding contrasts with data from high-income countries, where uterine rupture predominantly occurs in women with a previous cesarean section undergoing a trial of labor (9, 13). Similar proportions of rupture in unscarred uteri have been reported in African and Asian series, where multiparity, prolonged or obstructed labor, inadequate antenatal follow-up, and delayed referral remain major contributing factors (4, 10, 13, 18). These findings confirm that, in low-resource settings, uterine rupture is frequently associated with preventable intrapartum factors rather than uterine scarring alone.

Similar findings have been reported in several low- and middle-income countries. Astatikie et al. (21) reported high maternal and perinatal morbidity associated with delayed diagnosis and referral in Ethiopia. Rajaonarison et al. (22) observed that prolonged obstructed labor remained one of the leading causes of uterine rupture in Madagascar. Likewise, studies from sub-Saharan Africa and South Asia consistently report high rates of unscarred uterine rupture, delayed referral, and elevated perinatal mortality compared with high-income settings. These observations highlight persistent inequalities in access to emergency obstetric care and reinforce the need for improved referral systems and intrapartum monitoring.

Neglected uterine rupture appeared to be associated with poorer maternal and perinatal outcomes. Higher rates of hysterectomy, severe hemorrhage, and massive blood transfusion were observed, reflecting the consequences of delayed surgical intervention (5, 12, 14, 15). Perinatal mortality was markedly elevated in neglected cases, in agreement with previous studies showing that fetal prognosis largely depends on the interval between rupture and delivery (4, 5, 21). These results underline the crucial importance of early diagnosis and prompt referral to equipped obstetric centers. These observations are descriptive and should not be interpreted as evidence of independent associations because inferential statistical analyses were not performed.

An important and concerning finding in our study is the association between uterine rupture and the use of uterine fundal pressure during labor. Several reports suggest an association between fundal pressure and uterine rupture, particularly in multiparous women or in the presence of obstructed labor (22, 23). Despite the absence of proven benefit, this maneuver continues to be practiced in some settings and represents a clearly avoidable iatrogenic risk factor (8, 19). Its systematic elimination should be strongly emphasized in obstetric training and clinical protocols.

Instrumental vaginal delivery was recorded in 16.2% of cases. Although causality cannot be established in this retrospective study, operative vaginal delivery performed in the setting of prolonged or obstructed labor may contribute to excessive uterine stress and has occasionally been reported among women experiencing uterine rupture. Careful patient selection and adherence to evidence-based obstetric guidelines remain essential.

Uterine rupture on a scarred uterus remains a major concern, particularly in the context of labor trial after cesarean section. Previous studies have demonstrated increased risks associated with short interdelivery intervals, classical cesarean scars, and inappropriate labor induction or augmentation (14, 16, 24–27). Although vaginal birth after cesarean can be safely achieved in selected cases, strict adherence to clinical guidelines and continuous intrapartum monitoring are essential (9, 13).

Strengthening antenatal care, ensuring skilled attendance at birth, promoting systematic use of the partograph, and improving referral systems are essential to reducing both the incidence and severity of uterine rupture (5, 19, 20). Education and training programs targeting healthcare providers in peripheral facilities could facilitate earlier recognition of labor complications and reduce delays in care.

This study has several strengths, including its relatively long study period and the analysis of both scarred and unscarred uterine rupture. However, certain limitations must be acknowledged. The retrospective design may have resulted in incomplete data, and the single-center nature of the study may limit generalizability. Nevertheless, as a tertiary referral hospital receiving severe cases from a wide geographic area, our findings likely reflect the reality of uterine rupture management in similar low-resource settings.

Uterine rupture remains a serious and largely preventable obstetric emergency. Delayed diagnosis, inadequate intrapartum monitoring, and harmful obstetric practices—particularly uterine fundal pressure—are major determinants of poor maternal and perinatal outcomes. Addressing these factors through strengthened health systems, adherence to evidence-based guidelines, and efficient referral pathways is essential to reducing the burden of uterine rupture.

### Implications for clinical practice

#### Key preventive measures include

1. Enhanced antenatal care, especially in multiparous women.
2. Monitoring labor risk factors: obstructed labor, macrosomia, malpresentation, and previous cesarean and scarred uterus.
3. Avoiding fundal pressure and unmonitored oxytocin augmentation.
4. Prompt referral systems for peripheral centers to tertiary hospitals.
5. Training for obstetric emergencies, including conservative uterine repair.
6. Emphasize the importance of multidisciplinary care, involving an obstetrician, midwife, neonatologist, and anesthesiologist.
7. Avoid traditional home births.

#### Limitations

This study has several limitations. First, its retrospective design may have resulted in incomplete or inaccurate documentation of clinical variables. Second, some important neonatal variables, including Apgar scores and birth weight, could not be analyzed because of substantial missing data. In addition, the anatomical site of uterine rupture was not consistently recorded in medical charts. Third, the study was conducted in a single tertiary referral hospital and may therefore overrepresent severe or neglected cases, limiting the generalizability of the findings. Finally, the descriptive design and relatively limited sample size did not allow robust multivariable analyses to identify independent predictors of adverse outcomes. Consequently, no causal relationship can be inferred between the identified factors and adverse maternal or perinatal outcomes.

### Strengths

- Large cohort over seven years.
- Comprehensive data on neglected vs. non-neglected UR.
- First Moroccan series highlighting UR as public health challenge in unscarred uteri and fundal pressure–related complications

### Global health implications

The results underscore uterine rupture as an ongoing public health concern in low-resource environments. The majority of identified risk factors—including insufficient antenatal care, delayed referrals, and application of uterine fundal pressure—are preventable. Enhancing health system infrastructure, advanced facilities and monitoring, providing comprehensive training for healthcare providers in peripheral facilities, and applying evidence-based obstetric protocols have the potential to significantly mitigate the incidence and adverse outcomes associated with uterine rupture.

## Conclusion

This retrospective analysis highlights several factors frequently observed among women experiencing uterine rupture in our setting, including previous cesarean section, multiparity, inadequate antenatal care, delayed referral, and prolonged labor. The findings emphasize the importance of early identification and rigorous management of high-risk women, especially those with a history of uterine surgery, multiparity, or prolonged labor that is inadequately monitored. Preventing uterine rupture depends on heightened clinical vigilance, particularly in patients attempting vaginal delivery after cesarean section and in resource-limited settings, as well as prompt decision-making and immediate access to emergency obstetric care. It is also crucial to minimize the use of radical surgical procedures such as hysterectomy, given their serious reproductive and psychological consequences, which necessitate appropriate psychological support. An effective prevention strategy must include strengthening prenatal care, improving labor monitoring, ensuring the presence of qualified personnel, and educating patients about the options for vaginal delivery after cesarean section to reduce maternal and fetal morbidity and mortality while preserving women's quality of life and reproductive autonomy.

## Data Availability

The original contributions presented in the study are included in the article/Supplementary Material, further inquiries can be directed to the corresponding author/s.
